# Endocannabinoids in neuroendopsychology: multiphasic control of mitochondrial function

**DOI:** 10.1098/rstb.2011.0393

**Published:** 2012-12-05

**Authors:** Alistair Nunn, Geoffrey Guy, Jimmy D. Bell

**Affiliations:** 1 Metabolic and Molecular Imaging Group, MRC Clinical Sciences Centre, Imperial College London, Hammersmith Hospital, London W12 0NN, UK; 2 GW Pharmaceuticals, Porton Down, Salisbury, Wiltshire SP4 0JQ, UK

**Keywords:** mitochondria, endocannabinoid, hormesis, redox

## Abstract

The endocannabinoid system (ECS) is a construct based on the discovery of receptors that are modulated by the plant compound tetrahydrocannabinol and the subsequent identification of a family of nascent ligands, the ‘endocannabinoids’. The function of the ECS is thus defined by modulation of these receptors—in particular, by two of the best-described ligands (2-arachidonyl glycerol and anandamide), and by their metabolic pathways. Endocannabinoids are released by cell stress, and promote both cell survival and death according to concentration. The ECS appears to shift the immune system towards a type 2 response, while maintaining a positive energy balance and reducing anxiety. It may therefore be important in resolution of injury and inflammation. Data suggest that the ECS could potentially modulate mitochondrial function by several different pathways; this may help explain its actions in the central nervous system. Dose-related control of mitochondrial function could therefore provide an insight into its role in health and disease, and why it might have its own pathology, and possibly, new therapeutic directions.

## 1. Introduction

The plasma-membrane-based endocannbinoid system (ECS) was first ‘identified’ in the 1990s, when a cognate receptor for tetrahydrocannabinol (THC) was finally cloned. Since then, innate ligands have also been isolated, including anandamide (AEA) and 2-arachidonyl glycerol (2-AG). Thus, the ECS is another component of the arachidonic-acid-based signalling system. For the purposes of this study, it will be defined as comprising the cannabinoid type 1 and 2 receptors (CB 1/2), the transient receptor potential vanilloid-1 receptor (TRPV1R), AEA, 2-AG and anabolic and catabolic enzymes, as well as its effects on membrane structure and function, particularly around lipid rafts.

The ECS, like every ‘system’ in a living organism, has evolved to ensure the survival of the animal, and because of this, we believe that it must interact and modulate other systems—in particular, the mitochondrion. For instance, the ECS is known to modulate extracellular signal-regulated protein kinase (ERK), protein kinase B/mammalian target of rapamycin (Akt/mTOR), redox, cAMP, calcium and nitric oxide (NO); these are also pathways and moieties known to control mitochondrial function. It may also be able to directly affect mitochondrial function via membrane interaction.

By understanding a potential ECS–mitochondrial connection, we might be able to further explain the function of the ECS, especially how it might control cell fate. In particular, how there may be a very important multi-phasic dose effect that may help explain why it can be both friend and foe. Thus, in this study, we would like to guide the reader through a series of reasonably well-established facts, which would support our hypothesis that the ECS modulates mitochondrial function. This idea is based on a very simple concept: any change in plasma membrane dynamics is coupled to mitochondrial function.

## 2. Endocannabinoid system pathways common to mitochondrial control

**Figure 1.**
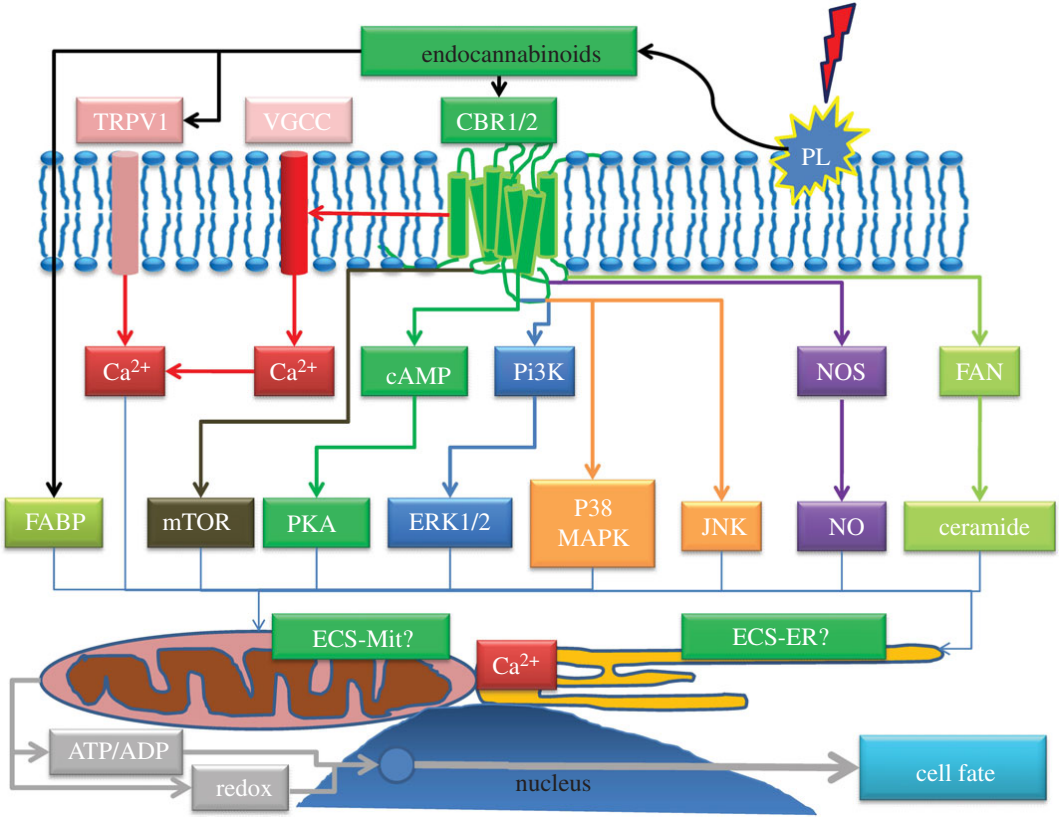
ECS pathways to mitochondrial control of cell fate. Pathways/signalling moieties known to both modulate the endocannabinoid system and mitochondrial function. CBR, cannabinoid receptor; ECS-ER, endocannabinoid system endoplasmic reticulum; ECS-Mit, endocannabinoid system mitochondria; ERK, extracellular receptor kinase; FABP, fatty-acid-binding protein; FAN, factor associated with neutral sphingomylinase; JNK, c-JUN N-terminal kinase; mTOR, mammalian target of rapamycin; NOS, NO synthase; PKA, protein kinase A; PL, phospholipase; TRPV1, transient receptor potential vanillioid 1; VGCC, voltage-gated calcium channel.

The ECS modulates many pathways and ions known to be involved in controlling mitochondrial function. This includes inhibition of voltage-gated Ca^2+^ channels and activation of inwardly rectifying K^+^ currents (Kir), MAPK, eNOS/iNOS (hence nitric oxide, NO) and PKA [1]. Cannabinoids can also modulate ceramide production [2], as well as mTOR [3–7]. Furthermore, endocannabinoids (and their derivatives) could also potentially modulate mitochondrial function directly. Figure 1 summarizes the key pathways.

Endocannabinoids are released ‘on demand’ from membranes—and are just one of many eicosanoid-based signalling pathways involved in inflammation, suppression of inflammation and resolution of injury [8,9]. They are released during exercise [10], fasting [11], by high fat diets [12] and certainly by damage—especially in the central nervous system (CNS), where they are thought to be neuroprotective [13]. They play a critical function, sometimes beneficial, and possibly detrimental, in ischaemia/reperfusion—and may have a preconditioning role [14].

At the organismal level, endocannabinoids increase appetite, but they also tend to reduce an organism's reproductive capacity by redirecting energy to storage [15]. They also reduce temperature and inhibit pain [16]. We have suggested that they modulate oxidative stress and energy metabolism (ensuring energy storage)—and could be described as ‘thrifty’[17]. Thus, like many biological systems, the ECS is generally beneficial, but can have its own pathology if inappropriately activated [18].

### (a) The endocannabinoid system could modulate mitochondrial function via calcium

Calcium is critical in controlling mitochondrial function and cell fate; however, mitochondria are also essential for controlling calcium flux [19]. The ECS reacts to (e.g. via calcium-activated phospholipases), and modulates, calcium flux in a number of ways (e.g. via mitogen-activated protein kinase (MAPK) and ion channels and intracellular calcium release). For instance, AEA can inhibit voltage-gated calcium channels, whereas AEA and 2-AG can increase intracellular free calcium [20]. Generally speaking, activation of TRPV1 tends to increase, whereas activation of CB1 tends to decrease intracellular calcium—but these two systems clearly interact and the response may vary between cells and be modulated by their status [21].

Endocannabinoids are generally seen as anti-proliferative and may induce apoptosis of cancer cells, mostly via CB1, but also by other mechanisms, such as via lipid rafts or cytochrome oxidase 2 [22]. In dorsal root ganglion neurons AEA activates TRPV1 allowing a calcium influx into the cell; this process is inhibited by concomitant activation of CB1—and may inhibit the sensitization process [23]. AEA can also induce cell death in cultured keratinocytes by increasing intracellular calcium, and the effect may require sequential activation of CB1, which then modulates the activity of TRPV1 [24]. In the brain, activation of CB1 by AEA at low doses reduces calcium influx, and has anxiolytic properties; however, it has now been suggested that at higher doses, it may activate TRPV1, which would induce calcium influx and have the opposite effect—being anxiogenic [25]. Intriguingly, recent data suggest that not only do CB1 receptors undergo endocytosis, but many receptors never make it to the plasma membrane but instead locate to endosomal and lysosomal compartments—where direct injection of AEA can release intracellular calcium [26]. This therefore suggests, at the broadest level, that there is a triphasic response involving modulation of calcium entry into the cell, and its release/sequestration within the cell; inhibition of calcium flux would effectively slow the cell down, a small increase could stimulate it (e.g. by mitochondrial production of hydrogen peroxide), while a big increase would tend to suppress mitochondrial function and eventually induce cell death—especially, if combined with other signals, such an increase in reactive oxygen species (ROS). There is a very tight link between ROS, calcium and ATP—mitochondrial overload with calcium, and excessive ROS, rapidly inhibit its function [19].

### (b) The endocannabinoid system could modulate mitochondrial function via nitric oxide

NO is an ancient signalling molecule, which modulates guanylate cyclase and thus, indirectly, mitochondrial biogenesis. It also directly regulates mitochondrial function by competing with oxygen at cytochrome c oxidase, the terminal acceptor in the mitochondrial electron transport chain (ETC)—so inhibiting energy production and increasing hydrogen peroxide. This activates AMPK (a key energy sensor in the cell) and can induce glycolysis: AMPK can also be activated by ROS and NO. The effect is to switch the cell from an anabolic to a catabolic status to conserve ATP. At higher concentrations, it starts to inhibit other components of the ETC, and can form peroxynitrite when combined with ROS and modulate the mitochondrial permeability transition pore (MPTP), and thus, cell death. The general consensus is that low levels produced by constitutive nitric oxide synthase (NOS), such as nNOS and eNOS (neuronal and endothelial), induce low-level cytoprotective mechanisms and mitochondrial biogenesis, whereas iNOS induces oxidative stress and is important in the inflammatory process. NOS activity is also modulated by calcium [27,28].

It has been known for some time that endocannabinoids can regulate NOS and NO production [29]. In a model of neurodegeneration, data suggest that CB2 activation increases nNOS in neurons, but decreases iNOS in astrocytes—and is thus anti-inflammatory [30]. Likewise, CB1 agonists can induce nNOS activation in neuronal cell lines [31], while low levels of AEA have been shown to stimulate eNOS in platelets via CB1 [32]. It has also been shown that AEA can induce NO production via TRPV1 in the endothelium [33]. iNOS is generally activated by proinflammatory mediators at normal intracellular calcium levels, whereas eNOS and nNOS require much higher calcium levels to become activated. iNOS tends to produce the highest levels of NO. In the brain, low-level NO production is vital for protection, whereas excessive NO production can become rapidly detrimental. This may be mirrored during nephropathy. Overall, this might suggest that targeting CB2 could be a useful clinical strategy [34].

The above suggest that among other pathways, NO levels, both absolute and probably in cellular context, play a vital role in controlling cell fate via modulation of mitochondrial function (among other effects, such as direct protein nitrosylation). For instance, a slight increase in NO may induce protective signalling in neurons, and possibly also induce slight increases in astrocyte glycolysis; this could be modulated via CB1 and CB2, and even TRPV1. If the insult was a bit larger, then it could lead to inflammatory levels of NO in some cells, but this could be offset by CB2 suppression of iNOS in inflammatory cells. If the signal was too large, mitochondrial function would be severely inhibited, leading to excessive necrotic cell death—which is inflammatory (apoptosis is normally a non-inflammatory process). Hence, there is potential for multiphasic effects, depending on concentration. However, the effects of NO would also be modulated by calcium levels, ROS, hydrogen peroxide and MAPKs.

### (c) The ceramide link

Many stress pathways also control mitochondrial function. One of the best described is based on ceramide; it can directly alter mitochondrial function by inhibiting the ETC, and can induce mitochondrial fission and opening of the MPTP. For instance, ceramide is released during ischaemia/reperfusion. It may even form a channel in the mitochondrial membrane [35,36]. However, data suggest that although many ceramides do have anti-proliferative and apoptotic effects, different chain lengths may actually have opposing effects, such as promoting cell survival. The effects may depend further on intracellular location and isoform [37,38].

Endocannabinoids may also modulate ceramide production. In lymphoma mantle cells, R (+) methanadamide (a stable analogue of AEA) increased ceramide levels and induced cell death; the effect involved activation of both CB1 and CB2 [39]. It is likely that their effects are highly context dependent in relation to other signalling but, critically, their actions can be explained by an integrated effect on the mitochondrion.

### (d) The endocannabinoid system and mitochondrial function could be coupled via redox

A critical point during evolution arose with the development of an oxygen-rich atmosphere, which provided an electron acceptor and enabled more energy to be released from food. As energy production relies on redox couples, and probably played a role in the evolution from single-celled to multicellular life [40], redox is critical to modern life—ranging from control of cell cycle, gene transcription, signal transduction, to immune cell function and (migration), both intracellularly and extracellularly [41–46]. Hence, it is no surprise that mitochondrial function is integrated with control of cell cycle—with redox playing a pivotal role [47]. However, none of this would be possible without membranes. Each part of a cell is compartmentalized for a different function, and each is kept at a different potential—with the plasma membrane being the most oxidized, and the mitochondrion the most reduced (followed by the nucleus). Within these compartments, the thioredoxin and glutathione systems maintain a dynamic redox balance that modulates cysteine status in multiple proteins. In this way, a cell can rapidly respond to any stress, as all these systems are coupled together [48,49].

Han *et al.* [50] have shown that CB1 and CB2 in macrophages may have opposing effects on ROS, with the former increasing, and the latter decreasing ROS and the production of pro-inflammatory cytokines. It therefore appears that excessive activation of CB1 in a tissue that is already inflamed appears to amplify the inflammatory cascade, which explains the benefits seen with antagonists in these conditions, while activation of CB2 appears to suppress this effect. The most recent example is in a model of cisplatin-induced nephropathy; pharmacological inhibition or genetic removal of CB1, greatly reduced AMPK activation and upregulation of nitrosative and oxidative stress and cell death [51]. It is therefore likely that the role of the ECS in relation to redox is contextual (disease versus normal state) and would be multiphasic. Activation of AMPK can upregulate PGC1α (PPARγ coactivator 1α) and induce mitochondrial biogenesis, as well activating autophagy of damaged mitochondria, or even inducing apoptosis if the stress is too great. The AMPK pathways sense energy decrease and stress, including calcium and ROS, and switch on catabolic pathways while switching off anabolic ones—such as mTOR [52].

A recent review of the role of the ECS in diabetes concluded that activation of the CB1 receptor was largely inflammatory and was associated with increased oxidative stress, while activation of the CB2 receptor had the opposite effect [53]. Certainly, it appears that CB1 activation in inflammatory states may amplify the ROS–MAPK pathway, so worsening the situation. The precise source of the AEA-induced ROS in endothelial cells was unclear [54]. However, data relating to propofol (an anaesthetic) may be of interest here; it partly protects HUVEC cells against AEA-induced ROS and death, possibly by virtue of its anti-oxidant properties [55]. Suggestively, propofol is also known to inhibit mitochondrial function by depolarizing the membrane, reducing mitochondrial membrane potential [56]. This might suggest that in part, mitochondrial ROS induced by AEA may be important. In this regard, it has been shown that 2-AG, via a mechanism possibly involving direct membrane absorption and not CN receptors, induces mitochondrial ROS production, which leads to the death of hepatic stellate cells—but not normal hepatocytes. The authors suggested that the difference was due to the levels of anti-oxidants in each cell [57]. In short, the response of a particular cell will depend on its anti-oxidant systems and mitochondrial function, in effect, its redox status. This therefore suggests that excessive activation of CB1 would become inflammatory in diabetes—quite possibly, by inducing severe mitochondrial stress. This could result in a vicious cycle, if dead cells were not cleared properly (i.e. they became necrotic).

### (e) The endocannabinoid system modulates mTOR: a key pathway in control of mitochondrial function

mTOR modulates mitochondrial function and controls lifespan; inhibiting mTOR tends to increase longevity and inhibit mitochondrial function, while activation tends to have the opposite effect [58,59]. Calorie restriction reduces mTOR activity, and may be one of the pivotal pathways involved in how it increases longevity [60]. It is now becoming clear that increased mitochondrial mass, and subsequent production of ROS and upregulation of anti-oxidant defences are generally associated with differentiation and eventual senescence, while decreased mitochondrial mass (and thus ROS), such as induced by hypoxia, is associated with ‘stemness’ in stem cells—indicating an improved ability for self-renewal and proliferation [61].

Despite much research showing that the ECS is often anti-proliferative, it is also involved in axonal path-finding, neural plasticity and neurogenesis [62]. It seems that CB2 receptors maybe primarily located on undifferentiated neural progenitor cells, and that activation of CB2 by HU308 induces proliferation via a mechanism involving mTOR [6]. Likewise, using specific agonists and antagonists of CB1 and CB2, Gomez *et al.* [5] found that activation of mTOR was involved in inducing differentiation of oligodendrocyte progenitor cells obtained from mixed glial cell cultures. Interestingly, THC may also activate mTOR in a CB1-dependent way in the hippocampus, an effect mimicked by the fatty acid amide hydrolase (FAAH) inhibitor, URB597 [4]. FAAH is the key in endocannabinoid degradation. By contrast, THC and JW-015 (a CB2 agonist) induced autophagy in a cancer cell line in a mechanism that involved upregulation of AMPK and suppression of the mTOR pathway [3]. Thus, at one level, the ECS is capable of inducing proliferation; activation of mTOR is associated with both mitochondrial activation and cell proliferation. The key here is that a small increase in hydrogen peroxide could stimulate proliferation in quiescent cells with a low mitochondrial mass and a highly reduced redox potential; this redox signal could come from both the mitochondria and MAPK (see below). In effect, low-level activation of stem cells by the ECS could be proliferative and would involve mitochondrial activation.

### (f) The endocannabinoid system modulates MAPK and cAMP: a link to mitochondrial network organization

Another aspect of mitochondria is that they can form reticular networks throughout the cell; indeed, ‘mitochondria’ means beads on a string. These networks constantly break-up and reform in response to cellular conditions, including during the cell cycle. It is likely that their function is intimately integrated with the endoplasmic reticulum (ER), especially in relation to calcium and redox signalling and stress. This function is combined with kinase activity, and kinases are modulated by redox.

One group called the MAPKs react to external changes to the cell. For instance, ERK 1&2 tend to be activated by mitogens (growth signals), whereas p38 and c-jun N-terminal kinase (JNK) are activated by inflammation and stress; ERK1/2, P38 and JNK can translocate to the mitochondrion, and redox plays a critical role in controlling these pathways [47,63]. It is now thought that fused mitochondrial networks can transmit redox signals throughout the cell [64], demonstrating just how important the membrane-redox couple is. The ECS is known to modulate MAPKs [1], so suggesting a possible role in controlling mitochondrial networks. This concept is reinforced by another well-known target of the ECS—the cAMP pathway [20]; this pathway is critical in controlling mitochondrial function, especially mitochondrial dynamics and networks in response to energy demands [65]. This might suggest that the ECS could play a role in maintaining mitochondrial networks to transmit redox signals throughout the cell.

## 3. Possible direct control of mitochondrial function by the endocannabinoid system

An important emerging paradigm is just how inter-connected the membrane system of cells is. In particular, how the ER/caveolae system interacts with the plasma membrane, and how the ER interacts with the mitochondria system—itself forming an intracellular reticular network. For instance, it is now well described that, via mitochondria-associated ER membrane (MAM) junctions, there is close communication between these organelles, with the ER delivering calcium to the mitochondria. This system might also be involved in the transport of cholesterol, ceramides, ATP and proteins as well as in proteasomal protein degradation and lipid droplet formation [66]. Furthermore, it is also becoming apparent that both the ER and the mitochondria also contain lipid rafts [67,68]. These are not unlike those found in the plasma membrane, and play very important roles in controlling mitochondria function during apoptosis (such as fission) [69]. Critically, some lipophilic anti-cancer agents appear to be able to induce plasma membrane lipid rafts to be internalized and transported directly to the mitochondria [70]. There is thus the potential not only for plasma-membrane-derived endocannabinoids to circulate within the cell, but also for them to be produced intracellularly and have activity via an intracellular ECS.

### (a) Intracellular eiscosanoid-based pathways

Research to date suggests that most of the eicosanoid-based signalling pathways are derived from the plasma membrane. However, there is now evidence that these pathways also exist in the mitochondria—and could, potentially, produce endocannabinoids [71]. It also appears that cannabinoid receptors are active within lysosomes and are important in controlling calcium flux involving the ER [26]. Extracellular endocannabinoids are rapidly taken up by the cell and converted to arachidonic acid by FAAH—a process that may involve both passive diffusion and facilitated transport [72]. Some evidence also suggests that fatty-acid-binding proteins (FABPs) may also be able to transport endocannabinoids, and could even explain how they may reach the nucleus to activate PPARα [73]. FABPs are ubiquitous and promiscuous proteins that carry lipophilic molecules around the cell (such as lipophilic xenobiotics), targeting them to various organelles, including the nucleus (to activate transcription factors), the mitochondria (for oxidation) and the ER for storage [74,75]. Interestingly, although it remains to be reproduced by other groups, Benard *et al.* [76] have suggested that not only does CB1 appear to exist in mitochondria, but that direct suppression of mitochondrial function by THC or WIN is lost in isolated mitochondria from CB1 knockout mice.

### (b) Endocannabinoids and their derivatives could directly alter mitochondrial function

Since the early 1970s, it has been suspected that THC (a phytocannabinoid) might inhibit components of the mitochondrial ETC [77]. More recently, data suggested that as well as THC, AEA and HU210 could also inhibit the function of isolated mitochondria [78]. AEA has also been shown by other groups to directly inhibit mitochondrial function; Catanzaro *et al.* found that it dose-dependently increases mitochondrial swelling and reduced cytochrome c release induced by calcium ions. These effects were independent of its target receptors and were paralleled by decreased membrane potential and increased membrane fluidity [79]. More recently, data suggested that AEA can inhibit ATP synthesis at the level of F0F1 ATP synthase at low micromolar concentrations [80]; 2-AG may also do this, possibly by altering membrane fluidity [81]. FAAH also appears to be located on mitochondria [82], which suggests that it could degrade endocannabinoids in this location.

It has been long known that fatty acids, other than being fuel, also directly modulate mitochondrial function—one way they do this is by uncoupling, acting as protonophores that can reduce ROS production in some circumstances [83,84]. Uncoupling is known to stimulate PGC1α transcription, the master controller of mitochondrial biogenesis, so suggesting a built in feedback mechanism to prevent excessive oxidative stress [85]. Arachidonic acid, in particular, has potent effects on isolated mitochondria. At high nanomolar concentrations, it stimulates mitochondrial coupling, but at low micromolar concentrations, it induces uncoupling (reduces ROS). As the dose increases, it progressively inhibits components of the ETC and induces release of H_2_O_2_ and, potentially, cytochrome c; it can induce mitochondrial permeability at high concentrations in the presence of ROS [83]. It therefore displays a triphasic effect. Critically, it also appears that stress induces release of arachidonic acid from mitochondria—for instance, excessive mitochondrial calcium influx [86]. Interestingly, arachidonic acid can protect glial cells against peroxynitrite toxicity [87], which would support its potential role as a protonophore. These data therefore hint that either endocannabinoids could be delivered to the mitochondria (e.g. via lipid rafts or FABPs) or, if the data from Benard turns out to be true, they might also be produced *in situ*. Either they or arachidonic acid could therefore modulate mitochondrial function directly.

## 4. The importance of dose in endocannabinoid system action can be explained by mitochondrial control

An old, but increasingly recognized paradigm in biology is the effect of dose, which is epitomized by the ‘hormetic’ biphasic adaptive response—whereby a low dose of a stressor, whether it is a plant compound, cold or exercise, stimulates adaptation to resist it better the next time round. In effect, this is preconditioning, where too high a dose becomes detrimental [88]. The above discussions clearly highlight how the output of multiple pathways can be integrated via the mitochondrion, with different signal strengths having different outputs—ranging from stimulus to cell death. In relation to the ECS, this might suggest that its effects (depending on the status of the cell) could well be triphasic and possibly even quadriphasic: submicromolar doses stimulate, while low micromolar doses protect by uncoupling. As doses rise, there could be a progressive inhibition of mitochondrial function eventually leading to cell death—which could either be apoptotic or necrotic.

A possible example of this effect is that AEA at concentrations greater than 25 μM induces excessive necrotic cell death in hepatic stellate cells, while at lower doses (<10 μM) it inhibits proliferation—an effect blocked by anti-oxidants [89]. The precise outcome for each cell could then be explained by their current redox status, and thus stage in the cell cycle, and the status of their ECS. The system could have both local and distant effects, and could transmit stress information in a dose-related fashion; it could therefore be said to have hormetic properties—low doses inducing adaptation and upregulation of cellular defence mechanism, while high doses induce cell death [17].

With regard to pathways, mild activation of MAPK, mTOR and increased calcium influx, especially in a low ROS or NO environment, might activate mitochondrial function—increasing ATP, generating hydrogen peroxide and stimulating proliferation and/or activation of function. A slightly different mix may inhibit mitochondrial function, for instance, neutral calcium change but increased NO and suppression of mTOR, which via retrograde signalling could stimulate mitochondrial biogenesis and differentiation. In other cells, this may actually induce glycolysis and increased stemness. At even higher levels, perhaps with activation of TRPV1 and JNK, the cell would experience inhibition of mitochondrial function and production of ROS, loss of ATP and influx of calcium, and suppression of mTOR—which could lead, depending on the type of cell, to quiescence, senescence or apoptosis and critically, if very active, necrosis. Mitochondrial function could be further modulated by direct action. Thus, the ECS could control mitochondrial function by multiple pathways.

**Figure 2.**
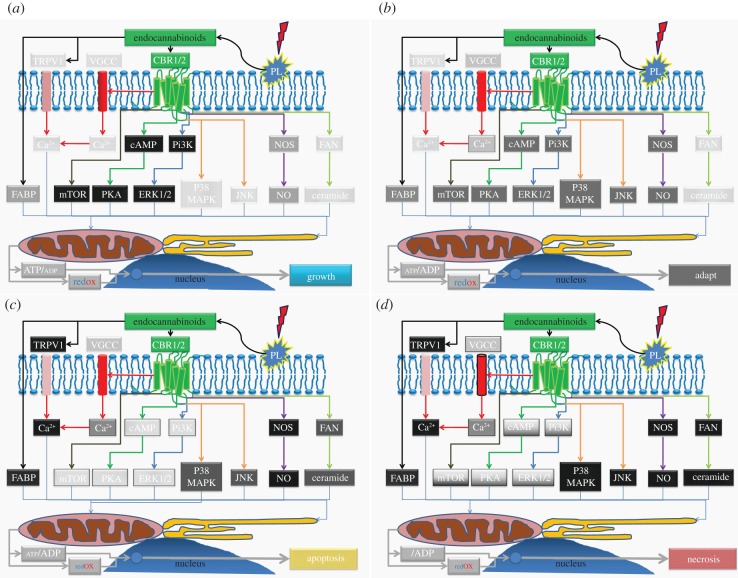
ECS dose–response-possible pathway activity. Pathways controlled by the ECS that can modulate mitochondrial function at different levels of activity. (*a*) Low-level stimulation—activates mitochondrial function and stimulates growth/proliferation pathways. Stress pathways at minimal activity. The increased mitochondrial activity in stem cells (which are highly reduced), triggers growth and would reinforce the hydrogen peroxide growth signal. In differentiated cells, increased mitochondrial activity would be associated with greater metabolism. Light grey indicates low activity of pathway, while black/darker grey indicates high activity. (*b*) Medium tone—starts to activate some stress pathways and suppress growth pathways, ensures adaptability—may induce stemness by upregulating anti-oxidant pathways. Some cells become slightly more oxidized, with a slight decrease in ATP—so would initially be anti-proliferative. Slight increase in mitochondrial calcium uptake, but increased ROS may act to suppress activity. Would activate AMPK and autophagy. (*c*) High tone—stress pathways are active and growth pathways switched off by increasing oxidative stress, but there is enough ATP production to ensure apoptosis. Cells becoming more oxidized and is associated with increased calcium and ROS production. (*d*) Excess tone—stress pathways would be fully active. The growth pathways may be switched on or off, depending on circumstances; hyper-activation would add to the problems. Mitochondrial function completely suppressed and not compatible with life. Very high oxidant levels and strongly inflammatory.

In summary, the ECS can be viewed as a stress response system, with four actions, depending on dose: proliferation, suppression and adaptation, apoptosis and potentially, necrosis—each of which could be explained by its actions on the mitochondrion. An overview of this can be seen in figure 2.

## 5. The endocannabinoid system as mitochondrial and energy guardian in the central nervous system

Neurons are highly differentiated and long-lived cells, and are very reliant on oxidative phosphorylation of glucose (although they can use ketones during starvation). Mitochondrial quality control is thus a critical part of maintaining their function—both by ensuring protein protection (e.g. by chaperones and proteases) and by continual fission/fusion. Fusion is critical in maintaining networks and exchanging genetic material and metabolites, and enhances ATP production. However, mitochondrial dysfunction can result in fragmentation and mitophagy, so removing damaged organelles and redistributing them. Indeed, a fall in mitochondrial membrane potential or matrix ATP can inhibit fusion [47]. Put simply, neurons are far more sensitive to mitochondrial dysfunction than most other cells, which is why their malfunctioning is associated with many neurodegenerative diseases [90,91].

Inflammation is a common factor in multiple CNS diseases, but it can suppress mitochondrial function. The reason is that inflammatory pathways induce an oxidative environment in the cell and act to suppress ATP production and metabolite production—these are ideal conditions to inhibit pathogen growth. Ultimately, it can induce cell death, so protecting the organism, as this environment is also damaging to the surrounding tissues [92,93]. As would be expected, mTOR function is inhibited during sepsis and inflammation [94]. Critically, sepsis itself can lead to widespread inhibition of mitochondrial function, potentially leading to energy crisis—a process triggered by inflammation-related oxidative stress [92]. However, inflammation is an energy-requiring process; activation of the inflammatory transcription factor, nuclear factor kappa B, increases energy expenditure [95]. In effect, inflammation displays the Warburg effect, whereby glycolysis is accelerated to provide metabolites for regeneration, although it is less energy efficient. Hence, in many ways, it has the opposite effect of ECS activation.

Under normal circumstances, inflammation is an evolved response to both cell damage and infection; the two usually (but not always) go hand-in-hand. If it works correctly, inflammation can lead to resolution of cellular/organ damage and infection, induction of proliferation of new cells, and removal of infected/damaged and front line professional apoptotic phagocytes by professional macrophages (and other phagocytes)—so minimizing excessive damage. Apoptotic cells tend to suppress inflammation, but necrotic cells stimulate it [96]. As resolution proceeds, there is a switch from Th1 to Th2 (type 1 to type 2 immunity), and suppression of phagocytic/inflammatory actions and a shift to humoral immunity; in effect, type 2 immunity is associated with resolution of cell-mediated immunity [97]. It is therefore of interest that under normal circumstances, the ECS appears to modulate a switch from a Th1 to a Th2 response [98]. It is thought that CB2 may play the most important role in neuromodulation, generally being anti-inflammatory—reducing activity of NO/ROS stress-inducing pathways. It is predominantly (but not exclusively) found in immune cells. However, as with CB1, if it becomes too active during inflammation, it can also induce further damage [99]. Data suggest that at least AEA might directly suppress NF-kB, the master regulator of inflammation, via CB2-dependent and CB2-independent mechanisms [100–102].

At the organismal level, inflammation is associated with the activation of the acute phase response, which ensures energy delivery to the brain and damaged tissues via insulin resistance, while initiating behavioural changes to ensure healing (thus, loss of libido, depression, anxiety, generally feeling unwell that ensure an animal lies down in a corner and conserves energy) [103]. This is known as cytokine-induced sickness behaviour and could be predicted from inhibition of mitochondrial function—especially in the brain. Hence, as normal activation of the ECS appears to be associated with anxiolytic activities, energy seeking and energy storage, suppression of Th1 inflammation and maintenance of neural function, it might be that the behavioural and metabolic aspects of the ECS almost diametrically oppose cytokine-induced sickness behaviour and associated metabolic changes. However, with one key similarity and one key difference, it would act to maintain energy supply to the CNS by maintaining a degree of insulin resistance and maintain oxidative phosphorylation—the most efficient use of energy. The above therefore suggests that under *normal circumstances*, the ECS is biased towards resolution of inflammation and repair of damage, adaptation to resist further insult, maintenance of neuronal mitochondrial function, as well as behavioural changes that ensure an animal feels better and seeks and replenishes energy stores—and keeps energy flowing to the brain. In effect, the ECS acts more on resolution from stress and counteracts mitochondrial dysfunction. In particular, maintenance of mitochondrial networks in long-lived neurons could be very important.

However, as would be indicated by figure 2, there may be well physiological circumstances where its over-activation might contribute to pathology. One interesting example may be the metabolic syndrome. This is a condition that may be caused by removal of nearly all factors from the environment that stimulate the production of efficient mitochondria and metabolic flexibility (‘hormetins’), such as physical activity, calorie restriction, temperature extremes and plant defence compounds. The result is a mitochondrially dysfunctional phenotype unable to deal with the almost unlimited supplies of calories available to many, which results in an inflammatory spiral and accelerated ageing [104]. The metabolic syndrome is thus associated with an increased prevalence of many diseases, such as cancer but, critically, it is also associated with increased rates of depression, which might be related to mild induction of cytokine-induced sickness behaviour [105].

Intriguingly, data also indicate that the ECS is either dysfunctional or over-active in the metabolic syndrome [106]. This might suggest that the ECS is responding to the inflammation and is a counter-regulatory system. This potentially means that the metabolic syndrome is a condition that has elements of both an injury and a resolution response, both of which, if highly active, might suppress mitochondrial function (probably in a tissue-specific manner)—but would ensure glucose supply to the brain. In the CNS, there is thus probably an ideal ‘Goldilocks’ zone where the right tone of the ECS maintains mitochondrial function and prevents excessive/inflammation. This might explain why antagonizing CB1 shows anti-metabolic syndrome effects, but is associated with CNS side-effects; in highly inflamed tissues, such as visceral adipose tissue (VAT) or the liver, it may tone down an over-heated ECS, but in the CNS, it reduces its protective function. Resolution may therefore require hormetins such as exercise or calorie restriction, and perhaps, even plant polyphenols that induce a widespread cellular hormetic response that breaks the cycle and restores homeostasis. Finding compounds, or combinations of compounds, that are both hormetic and antagonistic to an over-heated ECS may well provide therapeutic benefit—particularly if they are tissue-specific.

In summary, does the evidence support our hypothesis that the ECS must modulate mitochondrial function, and does it perhaps provide a deeper insight? Published data suggest that the ECS does modulate pathways and molecules well known to control mitochondrial function. Moreover, they support the emerging paradigm of biology that all systems are integrated and do not act in isolation, which allows for multiple levels of response and redundancy. However, it also displays a deep simplicity in relation to stimulus–response and adaptation to a varying environment—this was as true for our single-celled ancestors as it is for today's multicellular organisms. The mitochondrion is a single-cell symbiont in a modern cell that has enabled complex life to evolve; however, this complex life is still surrounded by a membrane and membrane signals need to be integrated with its function for adaptation and survival. The fact that the ECS controls multiple pathways that also modulate mitochondrial function cannot be a coincidence; it would be far more surprising if the ECS *did not* modulate mitochondrial function. We believe that data currently available support our hypothesis; in particular, it provides an insight into a stimulus–adaptation multiphasic response. Integration of membrane and mitochondrial function is a *sine qua non* condition of aerobic life in an ever-changing and challenging environment.
